# Iron deficiency anaemia: prevalence and associated factors among residents of northern Asir Region, Saudi Arabia

**DOI:** 10.1038/s41598-022-23969-1

**Published:** 2022-11-10

**Authors:** Tareg M. Belali

**Affiliations:** Faculty of Applied Medical Sciences, University of Bisha, 255, Al Nakhil, Bisha, 67714 Saudi Arabia

**Keywords:** Diseases, Health care, Risk factors

## Abstract

Iron deficiency anaemia is known to be one of the most common disorders that are associated with malnutrition. This study was conducted to form an understanding of the prevalence of Iron deficiency Anaemia (IDA) and evaluate its risk factors among the residents of the northern Asir Region, Saudi Arabia. Understanding the prevalence of IDA in different populations is important not only for therapeutic purposes but also for preventing the development of IDA in a given community. Moreover, this study was conducted to raise awareness about the significance of following iron-rich diet among high-risk groups such as women and children. This study collected data from 683 anaemic patients who are enrolled at the haematology unit in the Department of Internal Medicine at King Abdullah Hospital, Bisha Saudi Arabia. 398 participants who have IDA were included in this study where the collected data from the subjects included Age, gender, education, marital status, nationality, consanguinity, dietary habits and the clinical presentation of the participants. Our findings have shown that the prevalence of IDA among the participants is 58.27% where children under the age of 10 and females are the most affected individuals. Adults over 40, unmarried, and non-Saudis represented the second most affected portion of the subjects. IDA was prevalent among participants who shared the same ancestors and individuals with limited education. Moreover, participants did not consume sufficient iron and iron enhancing food or supplements. Inadequate iron intake is a major risk factor for anaemia. Low red fish and meat consumption contributed to the increase in ID. Findings highlight the need to raise awareness about the importance of a balanced diet and regular consumption of iron-containing food.

## Introduction

Anaemia is a clinical condition that is characterized by a reduction in the haemoglobin concentration with or without a reduction in red blood cell count^1^. Anaemias that are caused by nutritional deficiencies are a major concern worldwide; for instance, deficiencies in B12, iron, and folic acid could result in anaemias^1,2^. The WHO has identified Iron IDA as the most prevalent anaemia worldwide among children and pregnant women^2^. Moreover, they have reported that anaemia affects around 42% of children younger than 5 years and 40% of pregnant women, globally^2,3^. A reduced amount of iron in young children has severe consequences as iron is significant for mental development and its shortage could result in delays in the cognitive functions of a child. Iron deficiency (ID) can also impact the immune system, increasing a child’s vulnerability to Infectious organisms. In addition to that, an increase in the absorption of toxic metals can come from the high capacity for absorption among iron-deficient patients, which in turn may result in conditions such as lead poisoning and cadmium^5,6^. IDA in pregnant women could result in pregnancy complications such as easy fatigue, limited physical movement, lower cognitive abilities, and increased vulnerability to pyogenic infections. Moreover, pregnant women are at a higher risk of mortality as a result of severe ID. The foetus is also going to be at a high risk of stillbirth, premature delivery, neurological malfunction, and hypertension^6^.

Iron is considered to be one of the most important factors in cellular growth and differentiation^7^ and once the nutritional intake of iron is insufficient, the iron storage gets consumed and depleted fast to meet the body's requirements. Subsequently, a reduction in the haemoglobin synthesis occurs and the individual start developing anaemia where haemoglobin (Hb) concentration falls below the normal limits^8^. Given the history of IDA globally, it is paramount to observe that the nutritional shortage of iron is the main cause behind the development of IDA^9^. Subsequently, food distribution and public policies should be directed toward solving this problem in developing countries^10^. However, in Saudi Arabia, the epidemiological distribution of IDA is not well established and there are reports of IDA in rural areas and several institutions being around 10–60% of the overall anaemic patients^11^. Even though the prevalence of IDA is well studied in some areas of the country, there is a scarcity in the understanding of the prevalence of IDA and its risk factors in some of the rural areas in the northern Asir Region in Saudi Arabia.

### Objective

This study aims to estimate the prevalence of IDA among the residents of the northern Asir Region. The association of factors such as socio-demographics, lifestyle, and dietary habits to IDA was also studied.

## Methodology

### Study participants and sample size

The current observational study included 683 subjects. The data was collected using a survey that was conducted in 2022. After fulfilling the ethical criteria of the study set by IRB, the surveys were given to the patients who were enrolled at the haematology unit in the Department of Internal Medicine at King Abdullah Hospital, Bisha Saudi Arabia.

We followed the WHO criteria for IDA diagnosis that specified that an IDA subject's haemoglobin should be < 12.0 g/dL, combined with low serum iron and ferritin (serum iron < 10 μmol/L, serum ferritin < 15 μg/L) and low total iron binding capacity (TIBC ≥ 68 μmol/L). Subsequently, any patient who fits the criteria has been diagnosed with IDA. Pregnant women and individuals who suffered from chronic blood loss, gastrointestinal pathology, infections, and chronic inflammatory diseases were excluded from this study^4^. All participants contributed to this study of their own free will and after meeting the study's ethical standards for participation, the study was conducted under the tenets of the Declaration of Helsinki. The Institution Review Board of university of Bisha approved the study protocols. All participants gave written informed consent to participate in this study. The questionnaire distributed to 683 anaemic patients was collected and analyzed for this study.

### Study design

Two domains were considered in the sampling planning: (1) age (Under 10 years, 20–40 years, and above 40 years) and (2) gender (male or female). Three domains including education, marital status and nationality and consanguinity were also considered to form a sociodemographic background of the participants. The rest of the study domains aimed to collect data about the dietary habits and the clinical presentation of the participants.

### Ethical statement

This study was approved by the ethical Committee at IRB.

## Results

### Prevalence of IDA based on age, gender, and marital status

Out of the 683 anaemic patients, the prevalence of ID was 58.27% (398), while the rest suffered from sickle cell disease. A higher prevalence of IDA was observed among those aged under 10 years and ≥ 40 years and among females. There was no remarkable increase in the prevalence of anaemia among the participants aged between 20 and 40 years and males; however, they made up a significant portion of the participants (Table 1).

**Table 1:** Prevalence of iron deficiency among northern Asir Region residents according to gender and age.

Male	169
Female	229
Under 10 years	138
Above 40 years	135
20–40	125

### Distribution of IDA according to the socio-demographics of the participants

A noticeable increase in the prevalence of IDA was observed among the less-educated participants while the lowest prevalence was among the university-educated individuals. Non-Saudi participants represented the highest number of participants who suffered from IDA. Moreover, unmarried participants were also among those who were most affected by IDA. In addition, the findings suggest that consanguinity factors are related to the increase of IDA among the participants in this study. However, most of the participants did not have a familial history of IDA (Table 2).

**Table 2:** Frequency IDA based on sociodemographic factors.

Education	Primary	167
	Intermediate	219
	Secondary	8
	University and above	4
Nationality	Saudi	75
	Non-Saudi	323
Married		94
Unmarried		173
Consanguinity	Yes	212
	No	168
Past family history	Yes	164
	No	234

### Iron intake, dietary habits and clinical presentation of the participants.

This study has observed the adequacy of dietary iron consumption by the participants. The data about ingesting factors that could enhance or interfere with iron absorption was also collected. The findings suggest that the majority of the participants didn’t follow a specific regime. However, only a small fraction of the participants had any clinical symptoms or had received a blood transfusion (Table 3). Data regarding dietary intake shows that the majority of the study group consumed tea less than two times a week. The citrus intake, on the other hand, was low among the participants such that only 80 individuals reported ingesting citrus more than three times a week. However, poultry consumption was high among the participants and the majority of them consumed it more than three times a week. Insufficient meat and fish intake were observed among the subjects and the majority ate those less than two times a week (Table 4).

**Table 3:** Dietary regime, clinical presentation and receiving blood transfusion among the participants.

Participants following a dietary regime	Yes: 74
	No: 324
clinical presentation	Symptomatic: 78
	Asymptomatic: 320
Blood transfusion	Yes: 21
	No: 377

**Table 4:** Rate of dietary intake of tea, citrus, poultry, red meat and red fish among the participants.

Dietary intake		
Duration	≤ 2 times a week	≥ 3 times a week
Dietary & supplement		
Tea	229	169
Citrus	318	80
Poultry	112	286
Red meat	320	78
Red fish	381	17

## Discussion

IDA is a current important issue all over the world^12^. It is the most common nutritional anaemia in developing countries and it has become a major global health problem^4^. This study aims to estimate the prevalence of IDA and its associated factors among the residents of the northern Asir Region in Saudi Arabia. The findings of the present study show that there is a mild prevalence of IDA in the population of the northern Asir Region in Saudi Arabia. Nevertheless, children under the age of 10 and females are the most affected individuals by IDA. Adults over the age of 40, unmarried, and non-Saudis represented the population with a higher prevalence of anaemia. Furthermore, IDA was more prevalent in the participants who shared the same ancestors and individuals with limited education; meanwhile, the participants with higher education demonstrated the lowest number of participants with anaemia. The majority of the participants didn’t follow a clear dietary regimen but most of them consumed citrus—which facilitates iron absorption—less than two times a week. Tea consumption, which is known to interfere with iron absorption, was moderate among participants, where most of them had it less than three times a week. Poultry consumption was high among the participants while ingesting red fish and meat was low. Moreover, the majority of the participants never underwent a frequent blood transfusion and they exhibited only limited clinical symptoms.

The findings of the study are in agreement with the published literature in terms of the young children and females being the most affected by IDA. It ‏is prevalent among females due to multiple reasons such as lactation, menstrual bleeding, and pregnancy^6^. A previous study conducted at Taibah University, Saudi Arabia, on apparently healthy female students found that 171 out of the 268 participants had IDA^13^. However, another study that was conducted two years later found that there was a significant decrease in the prevalence of IDA (12.5%) among the female University students in Tabuk, Saudi Arabia^14^.

Familial history is a common factor in developing IDA as it has been described that IDA is prevalent among the relatives of β thalassemia patients^15^, which is in agreement with the findings of this study. However, the family history of IDA was found to be an independent risk factor by another study^16^, pointing out that the development of IDA in many cases could be attributed to nutritional deficiencies.

Correlating the educational levels of participants to the prevalence of IDA, it has been found that there is a low frequency of IDA among university-educated participants, which is in agreement with the findings of this study^17^. Unmarried individuals were found to be among the most who suffer from nutrition-induced IDA^18^. The lack of an iron-rich diet resulted in an increase in IDA among unmarried women, where 82% of the female participants were vegetarians^18^.

In agreement with the findings of this study, sufficient dietary iron intake and following a healthy dietary regimen are significant in eliminating iron deficiency. Food that contains diverse red meat and grains can reduce the risk of developing iron deficiency. Additionally, consuming iron supplements and iron-enhancing food eliminated the risk of the development of IDA in pregnant women. Consuming citric acid-rich food had positively impacted the status of IDA, where haemoglobin concentration remarkably increased when the study subjects consumed citric acid and lemon juice. Moreover, the regular consumption of citrus increases the bioavailability of iron and improves haemoglobin synthesis. However, based on the literature, the excessive consumption of tea inhibits iron absorption and results in ID. nevertheless, most of the participants in the present study consumed tea less than 2 times a week, therefore they might have developed IDA as a result of other factors. This is because the remarkable iron-binding abilities of the phenolic compounds with catechol groups in tea induce an inhibitory effect against the absorption of iron^19–23^.

## Conclusion

This study investigated the prevalence of IDA among the residents of the northern ASIR region, Saudi Arabia. This study identified inadequate iron intake as a major risk factor for anaemia. Excessive tea drinking habits, the low consumption of red fish and meat, and low ingestion of iron-enhancing food contributed to the increase of iron deficiency. The findings of this study highlight the necessity for raising awareness about the importance of a balanced diet and the regular consumption of rich sources of iron in daily meals. The results of this study contribute towards enhancing the level of understanding regarding the risk factors that contribute towards increasing the prevalence of IDA within the study area and help guide any future research that aims to address IDA.

## Data Availability

The data used to support the findings of this study is included within the article.
